# Award for outstanding contributions to the *Chinese Journal of Cancer*

**DOI:** 10.1186/s40880-015-0072-0

**Published:** 2015-12-23

**Authors:** Ji Ruan

**Affiliations:** Chinese Journal of Cancer, Sun Yat-sen University Cancer Center, Guangzhou, 510060 Guangdong P. R. China

## Abstract

At the 4th Guangzhou International Symposium on Oncology, Rui-Hua Xu, Chao-Nan Qian, and Wei Zhang—the chairmen and editors of the *Chinese Journal of Cancer*—announced and presented awards to 14 authors in recognition of their outstanding contributions to the journal.

Close collaborations between scientists and physicians from different backgrounds significantly improves our understanding of cancer biology. An excellent example of these collaborations was the 4th Guangzhou International Symposium on Oncology, which was held November 5–7, 2015, in Guangzhou, China. The Guangdong Anti-Cancer Association, the United States Chinese Anti-Cancer Association (USCACA), Sun Yat-sen University Cancer Center, and the *Chinese Journal of Cancer* (*CJC*) jointly organized this symposium. More than 1500 cancer researchers and clinicians attended this productive meeting, which included speakers from China, the United States, Italy, France, England, and Japan. The presentations covered several main types of cancer and reported progress in basic, translational, and clinical research.

The aforementioned organizations have jointly organized this symposium on oncology three times now. The first was the 2nd Guangzhou International Symposium on Oncology, held May 20–22, 2011, in Guangzhou, China [1]; the second was the 3rd Guangzhou International Symposium on Oncology, held November 20–22, 2013, also in Guangzhou, China [2].

At this the 4th Guangzhou International Symposium on Oncology, Rui-Hua Xu, Chao-Nan Qian, and Wei Zhang—the chairmen and editors of the *CJC*—announced and presented awards to 14 authors in recognition of their outstanding contributions to the journal in the past several years.

The *CJC* Editorial Board members selected the following 14 award winners.

1. Webster Cavenee, PhD
  Ludwig Institute for Cancer Research, University of California-San Diego, USA
2. Khurum Khan, MD, PhD
  Royal Marsden Hospital, England
3. Zhe-Sheng Chen, MD, PhD
  St. John’s University, USA
4. Takeo Nakanishi, MD, PhD
  Kanazawa University, Japan
5. Joseph Tien Seng Wee, MD, PhD
  National Cancer Centre, Singapore
6. Binhua P. Zhou, MD, PhD
  University of Kentucky School of Medicine, USA
7. Zhong-Zhen Guan, MD, PhD
  Sun Yat-sen University Cancer Center, P. R. China
8. Wan-Qing Chen, MD, PhD
  National Office for Cancer Prevention and Control/National Central Cancer Registry, P. R. China
9. Wen-Qi Jiang, MD, PhD
  Sun Yat-sen University Cancer Center, P. R. China
10. Wen-Lin Huang, MD, PhD
  Sun Yat-sen University Cancer Center, P. R. China
11. Zhi-Wei Zhou, MD, PhD
  Sun Yat-sen University Cancer Center, P. R. China
12. Su-Mei Cao, MD, PhD
  Sun Yat-sen University Cancer Center, P. R. China
13. Juan Wei, MD, PhD
  Department of Oncology, Second Affiliated Hospital of Southeast University, P. R. China
14. Yi Wan, MD, PhD
  Suzhou Kowloon Hospital, P. R. China

The guidance of the *CJC* Editorial Board members, the great contributions of reviewers, authors, and readers, and the tireless work of the editorial staff have resulted in papers published in the *CJC* being cited in many reputable journals. This has markedly increased the journal’s international impact. A testament to this came on July 22, 2014, when the *CJC* editorial office was notified by Thomson Reuters that *CJC* publications, beginning in 2012, had been accepted for coverage in the Science Citation Index Expanded, which is available at the Web of Science Core Collection, Biological Abstracts, and BIOSIS Previews [3]. The *CJC*’s first impact factor, obtained in June 2015, was 2.155.

The *CJC* will continue to highlight novel discoveries and technologies in all cancer research fields and relentlessly pursue the ultimate goal of eradicating this disease.
